# Oral Health Implications of GLP-1 Receptor Agonists and Other Incretin-Based Therapies

**DOI:** 10.3390/jcm15145358

**Published:** 2026-07-09

**Authors:** Julia Bijoch

**Affiliations:** Collegium Medicum–Faculty of Medicine, WSB University, 41-300 Dabrowa Gornicza, Poland; julia.bijoch@wsb.edu.pl

**Keywords:** GLP-1 receptor agonist, semaglutide, oral health, xerostomia, periodontitis

## Abstract

Incretin-based therapies have transformed the management of obesity and type 2 diabetes mellitus (T2DM). Established agents—the glucagon-like peptide-1 receptor agonists (GLP-1 RAs) semaglutide and liraglutide, and the dual GIP/GLP-1 receptor agonist tirzepatide—are now widely used, while a diverse pipeline of multi-receptor agents is advancing through late-phase development. GLP-1 receptors are expressed in several oral and other craniofacial tissues, raising the question of how these drugs affect oral health. This narrative review (PubMed, EMBASE, Scopus, Web of Science, Cochrane Library, ClinicalTrials.gov; January 2010–June 2026) integrates mechanistic studies, randomised controlled trials, pharmacovigilance data, society guidance, and the patient-facing phenomena increasingly raised in the dental chair. Preclinical and limited clinical evidence suggests that GLP-1 RAs may attenuate periodontal inflammation, while their effects on alveolar bone remain uncertain, reflecting both direct receptor and indirect metabolic influences. Concurrently, gastrointestinal adverse effects (nausea, vomiting, belching), reduced fluid intake, and possible salivary changes underlie a cluster of patient-reported complaints colloquially termed “Ozempic mouth”, “Ozempic teeth”, and “Ozempic breath”, alongside the facial soft-tissue changes seen in “Ozempic face”. Human oral-health-endpoint data remain scarce; much evidence is preclinical, indirect, or derived from case-level or pharmacovigilance reports. The oral cavity is nonetheless a plausible direct target of incretin pharmacotherapy and a practical monitoring site for tolerability. Dental practitioners should proactively manage the oral consequences of these agents, and validated oral health endpoints should be incorporated into future incretin trials.

## 1. Introduction

The global obesity epidemic, affecting more than one billion people, has driven an era of rapid pharmacological innovation [1]. Incretin-based therapies, which exploit the biology of glucagon-like peptide-1 (GLP-1) and glucose-dependent insulinotropic polypeptide (GIP), have become the leading pharmacologic strategy for obesity and type 2 diabetes mellitus (T2DM). In the pivotal STEP 1 trial, once-weekly semaglutide 2.4 mg produced a mean weight reduction of 14.9% at 68 weeks [2], and in SURMOUNT-1 the dual GIP/GLP-1 receptor agonist tirzepatide produced reductions of up to 22.5% [3]. The SELECT trial subsequently demonstrated a 20% relative reduction in major adverse cardiovascular events with semaglutide 2.4 mg in patients with obesity but without diabetes [4], extending the rationale for these agents well beyond glycaemic and weight management.

The oral cavity sits at the intersection of nutrition, metabolism, and systemic inflammation, yet it has received relatively little attention as a site affected by incretin therapy. GLP-1 receptors have been identified in rodent salivary gland tissue [5,6] and in periodontal ligament cells [7,8]. Obesity and T2DM are themselves major modifiable risk factors for periodontitis, salivary dysfunction, and impaired oral wound healing [9,10]. By mid-2024, approximately one in eight U.S. adults (12%) reported having ever used a GLP-1 RA [11]. Dental practitioners now routinely encounter such patients in clinical practice and are increasingly consulted regarding widely publicised effects such as “Ozempic mouth” and “Ozempic face”.

This review synthesises the evidence on how established incretin therapies (semaglutide, tirzepatide, liraglutide, dulaglutide) and the emerging multi-receptor pipeline (triple agonists, GLP-1/glucagon co-agonists, amylin-based combinations, GIP-antagonist hybrids—both injectables and oral therapies) intersect with oral health [12] and provides clinical recommendations for dental practitioners. Throughout, a careful distinction is drawn between mechanistically plausible hypotheses, preclinical findings, and demonstrated clinical effects, because the evidence base, while growing, remains immature.

## 2. Methods

This is a narrative review. PubMed, EMBASE, Scopus, Web of Science, the Cochrane Library, and ClinicalTrials.gov were searched for records published between January 2010 and June 2026, with the final search executed on 11 June 2026. Search terms combined drug-class descriptors (e.g., “GLP-1 receptor agonist”, “incretin”, “semaglutide”, “tirzepatide”, “retatrutide”, “cagrilintide”, “amylin”) with oral-health descriptors (“periodontitis”, “xerostomia”, “saliva”, “dental caries”, “tooth erosion”, “oral microbiome”, “dental implants”, “halitosis”). A representative full database search string is given here for transparency; as an illustrative example, the PubMed strategy combined (“glucagon-like peptide-1 receptor agonist” OR “GLP-1” OR “incretin” OR “semaglutide” OR “tirzepatide”) AND (“oral health” OR “periodontitis” OR “xerostomia” OR “saliva*” OR “dental caries” OR “tooth erosion” OR “halitosis” OR “dental implant*”), without language restriction, with the strategy adapted to the syntax of each database. The asterisk (*) denotes a truncation (wildcard) operator that retrieves all word endings sharing the preceding stem (for example, “saliva*” retrieves saliva, salivary, and salivation). Reference lists of retrieved articles, relevant society guidance (American Society of Anesthesiologists; consensus periodontology statements), and, given the prominence of patient-facing terminology, representative lay and clinical commentary on “Ozempic mouth”, “Ozempic teeth”, “Ozempic breath”, and “Ozempic face” were additionally screened to identify the patient-facing phenomena and terminology. These phenomena were then, wherever possible, re-grounded in peer-reviewed sources, and any observation resting on commentary alone is treated as hypothesis-generating rather than as evidence. Because dedicated oral-health-endpoint trials of incretin agents are largely absent, much of the human evidence presented is indirect (derived from metabolic trials) or preclinical. The strength of evidence for each clinical recommendation is labelled using a simplified GRADE-informed scheme (see Section 11). Peer-reviewed primary studies, systematic and narrative reviews, and formal society or regulatory guidance were eligible for inclusion where they addressed incretin pharmacology, GLP-1/GIP receptor biology in oral or craniofacial tissue, or an oral health endpoint relevant to the agents under review. Because this is a narrative rather than systematic review, citation was purposive rather than exhaustive: among peer-reviewed sources, priority was given to randomised controlled trials (RCTs), mechanistic and translational studies, and contemporary consensus statements, with older or lower-tier sources cited only where they remained the best available evidence for a given point. Where patient-facing phenomena were initially identified through lay or clinical commentary, peer-reviewed sources (narrative reviews, case series, and pharmacovigilance analyses) were sought and substituted wherever available, so that clinical claims rest on peer-reviewed evidence; the small number of remaining non-peer-reviewed items (for example, a usage-prevalence figure drawn from a survey news release) are used only for contextual or terminological purposes, not for efficacy or safety claims. No formal PRISMA flow diagram was generated, consistent with the narrative design.

## 3. Incretin Pharmacology and the Multi-Receptor Landscape

### 3.1. GLP-1 and GIP Physiology

GLP-1 is a proglucagon-derived peptide secreted from intestinal L-cells; its principal actions are glucose-dependent insulin secretion, glucagon suppression, delayed gastric emptying, and central appetite suppression. Native GLP-1 is degraded within minutes by dipeptidyl peptidase-4 (DPP-4); therapeutic agents are therefore structurally modified to resist degradation and to achieve a prolonged half-life [13]. GIP, secreted from duodenal K-cells, also potentiates insulin secretion, and the co-targeting of the GIP receptor (GIPR) and GLP-1 receptor (as in tirzepatide) yields metabolic effects that exceed those of selective GLP-1 agonism [14]. The GLP-1 receptor is a class B G-protein-coupled receptor that signals chiefly through the cyclic adenosine monophosphate–protein kinase A (cAMP–PKA) pathway, and its expression in extra-pancreatic tissues underlies the broad physiological reach of these agents [13].

### 3.2. A Mechanism-Based Taxonomy of Incretin and Incretin-Adjacent Agents

Rather than cataloguing brand names, it is more useful, particularly when reasoning about oral tissue effects, to organise agents by the receptors they engage. Each receptor combination carries a distinct physiological signature that may translate into distinct oral consequences. The principal validated and investigational classes are summarised in Table 1.

The practical message of this taxonomy for the dental reader is that oral health evidence exists almost exclusively for GLP-1 mono-agonists; for every other class in Table 1, oral effects are inferred from receptor expression and mechanism rather than demonstrated. As multi-receptor agents reach the clinic, each newly engaged receptor (glucagon, amylin/calcitonin, GIP-antagonism) introduces a distinct and currently untested set of possible oral consequences.

### 3.3. The Emerging Multi-Receptor Frontier and Its Untested Oral Implications

The agents now reshaping the field engage receptors beyond GLP-1, and each additional target remains, from an oral health standpoint, uncharted. Retatrutide, a GIP/GLP-1/glucagon triple agonist, produced a mean weight reduction of 24.2% at 48 weeks in its phase 2 trial—the highest value reported to date for any pharmacologic agent—and has advanced to the phase 3 TRIUMPH programme. Its glucagon receptor component adds thermogenic and hepatic effects, and its gastrointestinal and emetic burden during dose escalation is expected to equal or exceed that of currently available agents, which would, in turn, heighten the erosive and cariogenic risks discussed below [15]. The glucagon co-agonists (survodutide and mazdutide), the GIP-antagonist/GLP-1 bispecific agent maridebart cafraglutide, the amylin analogue cagrilintide and its semaglutide combination CagriSema (with weight loss of approximately 15.7–22.7% across the REDEFINE programme) [16,17], and the unimolecular GLP-1/amylin agent amycretin each engage receptor systems—glucagon, GIP antagonism, and amylin/calcitonin signalling—that are expressed in, or relevant to, oral tissue but for which essentially no oral health literature exists [12]. The practical implication is that the oral safety profile of each next-generation agent must be regarded as unknown until specifically investigated, and should not be assumed to mirror that of the GLP-1 mono-agonists. It should be stressed that, for every agent beyond the GLP-1 mono-agonists, the statements above regarding gastrointestinal burden and oral relevance are mechanistic extrapolations from receptor pharmacology, not observations; no oral health data exist for these compounds, and the inferences are offered as hypotheses to be tested rather than as anticipated clinical effects [12].

### 3.4. GLP-1 Receptor Expression in Oral and Craniofacial Tissues

GLP-1 receptor expression has been demonstrated by RT-PCR and immunohistochemistry in the parotid, submandibular, and sublingual glands of rodents, with receptor localisation to ductal and acinar compartments [5,6], and submandibular GLP-1R expression appears to be altered in the diabetic state [6]. Functional GLP-1R has also been confirmed in human periodontal ligament cells, where receptor activation modulates inflammatory signalling [7,8]. Whether these receptors are present and functionally significant in the corresponding human salivary tissues at therapeutically relevant drug concentrations has not been definitively established and should be regarded as an open question rather than settled fact.

## 4. Salivary Glands, Dry Mouth, and Salivary Composition

Saliva is essential for lubrication, antimicrobial defence, pH buffering, enamel remineralisation, and taste [18]. The demonstration of GLP-1R in salivary tissue [5,6] raises the possibility that GLP-1 RAs directly modulate salivary secretion; however, direct measurements of human salivary flow rate remain limited. Dry mouth is reported during the clinical use of these agents, and the dental literature consistently identifies xerostomia as a prominent complaint [19]. Importantly, the reduction in salivary output observed in GLP-1 RA users is likely to be multifactorial, combining any direct glandular effect with a marked reduction in fluid intake (patients report reduced thirst and food intake) and nausea-related changes in oral behaviour; the relative contribution of each factor remains unquantified [20,21].

Reduced salivary flow has downstream consequences: impaired buffering and remineralisation raise caries risk, and a drier oral environment favours odour-producing and cariogenic bacteria. These mechanistic links are well established for xerostomia of other aetiologies [18]; what remains uncertain is the magnitude and reversibility of any GLP-1 RA-specific salivary effect, which prospective sialometric studies will be required to define.

A 2025 mechanistic review has proposed a molecular framework for this effect, suggesting that semaglutide’s strong albumin binding, by prolonging receptor occupancy, may disturb the rhythmic calcium and cyclic AMP cross-talk required for normal salivary secretion, with β-arrestin-mediated receptor desensitisation and internalisation further reducing glandular responsiveness over time. Consistent with a possible agent-specific signal, pharmacovigilance analyses report dry mouth disproportionately more often for semaglutide (reporting odds ratio 3.21) than for liraglutide (1.80) or exenatide (1.26), and a small case series has described severe hyposalivation with an onset of approximately four weeks after initiation [21]. These observations remain hypothesis-generating, since spontaneous-report databases cannot establish causality and no direct human salivary pharmacodynamic data yet exist; they nonetheless reinforce the case for prospective sialometric study. A recent dental review of semaglutide-related oral effects further notes that sustained hyposalivation predisposes to root caries on exposed root surfaces in older patients and to an increased risk of oral candidiasis [22].

## 5. Periodontitis: Mechanisms and Emerging Evidence

Periodontitis is a chronic, dysbiosis-driven inflammatory destruction of the tooth-supporting apparatus and a major global disease burden [1]. Its relationship with T2DM is bidirectional: diabetes worsens periodontal destruction, and periodontitis, through systemic inflammatory mediators, impairs glycaemic control [23]. Critically, periodontal treatment improves glycaemic control, with meta-analyses reporting glycated haemoglobin (HbA1c) reductions of roughly 0.4% [24]. Obesity independently amplifies periodontal inflammation through adipokine dysregulation and immune dysfunction [9,25]. Because GLP-1 RAs act on all three of these drivers—hyperglycaemia, adiposity, and systemic inflammation—there is a coherent rationale for a potential periodontal benefit.

The direct periodontal effects of these agents have so far been characterised almost entirely in the laboratory. In vitro, GLP-1 RAs (including liraglutide and exenatide) promote the osteogenic differentiation of periodontal ligament stem cells through Wnt/β-catenin and mitogen-activated protein kinase (MAPK) signalling, even under high-glucose or inflammatory conditions [8,12]. In a lipopolysaccharide-stimulated human periodontal ligament cell model and in animal periodontitis models, liraglutide reduced inflammatory bone destruction and exerted anti-inflammatory effects [8]. A 2025 narrative review synthesising ten in vitro studies, nine preclinical animal studies, and a single clinical study concluded that GLP-1 RAs may improve periodontal and peri-implant health, while emphasising that clinical evidence is minimal and that the field rests largely on laboratory and animal data [12].

This preclinical case is substantive, but it must be weighed against the limits of the underlying evidence. No adequately powered randomised controlled trial has yet tested a GLP-1 RA against an active comparator with periodontal endpoints as the primary outcome while controlling for glycaemic change. Claims that these agents “treat” or “ameliorate” periodontitis are therefore premature; the evidence supports only the conclusion that benefit is biologically plausible and preclinically supported, and that definitive trials are needed.

It is important to distinguish the possible pathways through which any human periodontal benefit might arise, because they carry different clinical implications. A direct pharmacologic effect would reflect GLP-1 receptor signalling in periodontal ligament and immune cells, as suggested by the in vitro and animal data above [7,8]. An indirect effect, by contrast, could follow entirely from the metabolic and behavioural consequences of treatment, namely, weight loss, improved glycaemic control, improved diet, and reduced systemic inflammation, each of which independently benefits the periodontium [9,25]. Existing human observations cannot separate these pathways, and an apparent periodontal improvement during incretin therapy should therefore not be attributed to a direct drug effect. Until HbA1c-matched trials with periodontal primary endpoints are available, periodontal benefit remains a hypothesis, and periodontal care should continue to follow established risk-based standards independent of incretin use [12]. In practical terms, the clinical implication of this distinction is that incretin therapy should not prompt any relaxation of periodontal diagnosis, treatment, or recall intervals, and clinicians should neither defer indicated periodontal therapy nor lengthen maintenance schedules in the expectation of a drug-mediated benefit; the prudent default is to treat the periodontium exactly as one would in any patient at equivalent risk, while remaining alert to—but not assuming—a possible adjunctive effect that future trials may or may not confirm.

Periodontitis and cardiovascular disease share inflammatory pathways, and periodontal pathogens contribute to systemic inflammation [26]. Given the cardiovascular benefit demonstrated in SELECT [4], it is reasonable to hypothesise, though not yet to assert, that some fraction of incretin cardiovascular benefit could involve the attenuation of periodontal–systemic inflammatory signalling. This hypothesis warrants dedicated periodontal substudies within cardiovascular outcomes trials.

## 6. “Ozempic Mouth”, “Ozempic Teeth”, “Ozempic Breath”, and “Ozempic Face”

Several terms coined by patients and amplified in the media—“Ozempic mouth”, “Ozempic teeth”, “Ozempic breath”, and “Ozempic face”—are increasingly raised by patients during dental consultations. None constitutes a formal clinical diagnosis, and high-quality data remain limited; nevertheless, each maps onto a biologically plausible mechanism, and dental practitioners should therefore be prepared to address them candidly and accurately. “Ozempic mouth” and “Ozempic teeth” are umbrella terms for a cluster of oral complaints reported by patients on GLP-1 RAs: dry mouth, a sticky or coated tongue, tooth sensitivity, enamel erosion, gum irritation, and, in some accounts, the sudden appearance of cavities or even loosening teeth in patients previously stable [19,27]. Clinicians describing these cases attribute them to a convergence of mechanisms: reduced saliva (from diminished fluid intake and possible direct glandular effects), acid exposure from vomiting and reflux, and altered eating behaviour [27,28]. Nausea and vomiting are common during dose escalation, and repeated exposure of enamel to gastric acid produces the erosive pattern (notably on palatal surfaces of upper teeth) characteristic of intrinsic acid wear [2,3,19]. To prevent these patient-facing terms from being read as validated adverse-effect syndromes, and to make explicit the differing strength of evidence underlying the phenomena discussed here and in the salivary (Section 4) and caries/erosion (Section 7) sections, the evidentiary basis for each is summarised in Table 2. That table assigns every phenomenon to one or more explicit categories—direct human data, pharmacovigilance signal, preclinical mechanism, extrapolation from the general xerostomia or erosion literature, and expert or media/patient report—so that anecdotal or media-amplified observations are not conflated with established dental pathophysiology.

“Ozempic breath” refers to halitosis reported by some users. It is not listed in the prescribing information, and few peer-reviewed data specifically link these agents to oral malodour; current commentary emphasises that further study is required before firm conclusions can be drawn [19,29]. Three mechanisms are most often invoked. First, belching (“eructation”, the “Ozempic burp”) is a documented adverse effect of semaglutide, reported in roughly 7% of treated patients in pooled trial and labelling data [20]. Delayed gastric emptying may also allow gastric contents to ferment, releasing sulphur-containing gases [20]. Second, reduced salivary flow permits the proliferation of anaerobic, odour-producing bacteria on the posterior dorsum of the tongue—the principal source of physiological halitosis through volatile sulphur compound production. Reduced flow also diminishes the natural cleansing of the tongue, producing the “coated tongue” appearance reported in the dental literature [19]. Third, the metabolic shift towards fat utilisation (ketosis) during rapid weight loss can contribute a distinct breath odour [29]. A common patient question concerns reversibility. The answer differs according to the component considered. Dry mouth, taste changes, and breath effects are plausibly reversible as gastrointestinal effects attenuate or on discontinuation, and clinicians note that many oral symptoms improve as the body adjusts [29]. Structural tooth damage is not: enamel erosion and established carious lesions are permanent, and the dental literature notes that tooth damage may not reverse even if the medication is stopped [27]. This asymmetry is the central clinical message: preventive dental care during therapy is essential precisely because the irreversible component (hard-tissue loss) cannot be recovered later. “Ozempic face” describes the gaunt, hollowed, or prematurely aged facial appearance some patients develop after rapid, large-magnitude weight loss [12]. It is not specific to semaglutide—any rapid, large-magnitude weight loss can produce it—but the scale and visibility of the GLP-1 RA-treated population have brought it to prominence. Facial fat compartments (buccal, malar, periorbital) provide structural support; with weight loss exceeding 15–22% of body weight, as seen in STEP and SURMOUNT [2,3], these compartments deflate faster than overlying skin can contract, producing laxity, deepened folds, and temporal and cheek hollowing, particularly in older patients. Although the broader craniofacial and aesthetic dimensions of this change lie outside the scope of an oral health review, it is included here only because of its direct consequences for oral rehabilitation; the discussion is therefore confined to those dental sequelae and does not address aesthetic management as such. For dental practice, the relevant consequences are practical. Loss of facial soft-tissue volume and ridge changes accompanying weight loss can alter the fit of dentures and removable prostheses, necessitating earlier relining or remaking. Reduced buccal and masseteric soft-tissue bulk may also alter occlusal perception and jaw function, although this last possibility is a mechanistic hypothesis that has not been formally studied and requires confirmation. From a dental standpoint, the practitioner’s role is limited to recognising the change, counselling the patient that it is a predictable consequence of successful weight loss rather than a sign of oral disease, and, if the patient is concerned about facial appearance, directing them to the appropriate medical or specialist service; the aesthetic management of facial volume loss itself lies outside the dental remit and the scope of this review [12].

## 7. Caries and Erosion

The caries and erosion risks introduced by GLP-1 RAs follow directly from their side-effect profile. Vomiting exposes enamel to gastric acid (pH well below the critical demineralisation threshold of ~5.5), producing erosive wear [2,3,19], while reduced salivary flow impairs the buffering and remineralisation that normally protect against acid challenge [18]. Behaviourally, low-volume carbohydrate “grazing” to manage nausea provides frequent fermentable substrate for cariogenic bacteria such as *Streptococcus mutans* and *Lactobacillus* species [19]. It should be emphasised that these are well-established pathophysiological mechanisms extrapolated from the general erosion and caries literature; the erosive and cariogenic processes themselves are not in doubt, but their incretin-specific incidence and magnitude have not been quantified, and the relevant evidence category is indicated in Table 2.

These risks are partly offset by countervailing factors. Improved glycaemic control lowers cariogenic salivary glucose and improves oral immune function [25], and overall caloric and carbohydrate reduction may decrease total fermentable substrate. The net effect in any given individual depends on the balance among vomiting frequency, salivary change, dietary behaviour, and baseline risk, which argues for an individualised, rather than a uniform, approach to caries risk classification.

## 8. The Oral Microbiome

Obesity is associated with oral microbial dysbiosis that parallels systemic inflammation, and a drier oral environment shifts the microbial community towards odour-producing *P. gingivalis,* and cariogenic species such as *S. mutans* and *Lactobacillus* spp. [9,19]. Intriguingly, the relationship between the periodontal microbiome and incretin biology may be bidirectional: certain periodontopathic bacteria, notably *Porphyromonas gingivalis*, express dipeptidyl peptidase-4 (DPP-4)-like enzymes capable of degrading GLP-1, providing a putative microbial mechanism by which periodontitis could impair host incretin signalling and glucose regulation [30,31]. Whether GLP-1 RAs beneficially remodel the oral microbiome—for example, by reducing gingival crevicular glucose, attenuating inflammation, or altering salivary antimicrobial proteins—is mechanistically plausible but has not been established by robust longitudinal human metagenomic data [12]. This represents a clear and tractable research gap: prospective oral microbiome profiling before and during incretin therapy, paired with metabolic endpoints, would directly test these reciprocal hypotheses.

## 9. Alveolar Bone and Dental Implant Outcomes

GLP-1 RAs have complex effects on bone. In preclinical settings, receptor agonism promotes osteoblast activity and may restrain osteoclastogenesis, suggesting direct anabolic and anti-resorptive potential. Clinical data, however, are less encouraging: in a phase 2 randomised trial in adults at increased fracture risk, 52 weeks of once-weekly semaglutide was associated with increased bone resorption and lower bone mass at the lumbar spine and total hip versus placebo, changes attributed largely to reduced mechanical loading following weight loss rather than to a protective drug effect [32]. Whether incretin therapy is, on balance, protective or detrimental for the alveolar bone—one of the most metabolically active skeletal sites—therefore remains genuinely uncertain, and is likely to depend on the balance between any direct receptor-mediated effect and the skeletal consequences of rapid weight loss.

For dental implants, where local osseointegration rather than systemic bone mass governs outcomes, patients with obesity and T2DM face elevated failure and peri-implantitis risk [10]. Preclinical and limited clinical evidence summarised in the 2025 periodontal/peri-implant review suggests GLP-1 RAs may improve osseointegration and peri-implant health, plausibly via the osteogenic differentiation of periodontal ligament stem cells observed in vitro [8,12], but prospective implant outcome trials in incretin-treated patients are lacking. Until such data exist, GLP-1 RA use is best regarded as a possible favourable modifier rather than an established one.

## 10. Perioperative and Sedation Considerations

Because GLP-1 RAs delay gastric emptying, they raise concern for retained gastric contents and aspiration during sedation or general anaesthesia, a consideration directly relevant to dental procedures requiring deep sedation or general anaesthesia. Guidance has evolved. The 2023 American Society of Anesthesiologists (ASA) consensus recommended holding weekly agents for one week and daily agents on the day of the procedure [33]. In October 2024, updated multi-society guidance (ASA with the American Gastroenterological Association, the American Society for Metabolic and Bariatric Surgery, and others) shifted towards a more permissive, risk-stratified stance: most patients may continue their agent, with higher-risk patients following a 24 h liquid diet, anaesthetic plan modification, and point-of-care gastric ultrasound where concern exists. Dental practitioners arranging sedation should therefore coordinate with the anaesthesia team and prescriber and follow current institutional protocols, recognising that the evidence base for any specific holding interval remains incomplete [34].

## 11. Clinical Recommendations for Dental Practitioners

Recommendations are graded using a simplified GRADE-informed scheme: (A) supported by randomised controlled trial evidence; (B) supported by observational/cohort evidence or expert consensus with mechanistic support; and (C) expert opinion or extrapolation from analogous conditions. Given the immaturity of the oral health evidence base, no recommendation reaches Grade A; all are Grade B or C, and are labelled as such explicitly rather than overstating certainty. The recommendations detailed below are summarised in Table 3. Several Grade B recommendations (notably those for caries/erosion prevention, dry-mouth management, and breath) derive their support from the general dental literature on erosion, xerostomia, and physiological halitosis rather than from direct evidence in incretin-treated dental patients; this basis is stated explicitly in the “evidence basis/rationale” column of Table 3 so that the grade is not read as implying incretin-specific clinical data. These recommendations are graded B, rather than C, because the extrapolation is not a bare analogy: it is anchored in a defined, drug-related mechanism (treatment-emergent vomiting, reduced fluid intake, and delayed gastric emptying) together with indirect human data from metabolic trials and pharmacovigilance, which together constitute the “mechanistic support” required for Grade B under the scheme above. Grade C is reserved for recommendations resting on expert opinion or analogy alone, without such a mechanistic link. Where a measure carries greater specificity than the direct evidence supports, it is intended as a pragmatic, low-risk preventive default rather than an evidence-based threshold.

### 11.1. Medication History

At every examination and recall, incretin agent use (name, dose, duration, and tolerability, especially nausea/vomiting and belching frequency) should be documented because patients seldom volunteer this information and the oral consequences cluster early in therapy [Grade C].

### 11.2. Caries and Erosion Prevention

Stratify caries risk individually, escalating for patients with frequent vomiting or carbohydrate grazing. Inspect the palatal surfaces of the maxillary teeth in particular for early erosion—the pattern characteristic of intrinsic acid from vomiting (perimolysis)—with occlusal surfaces affected secondarily. Counsel patients to rinse with water or a sodium bicarbonate solution after vomiting and to defer brushing for 30–60 min to avoid abrading acid-softened enamel [35] [Grade B].

- High-fluoride (5000 ppm) toothpaste for moderate- to high-risk patients [35].
- Fluoride varnish 2–4 times yearly; neutral sodium fluoride rinse for erosion-prone patients [35].
- Dietary counselling to reduce frequency of fermentable carbohydrate exposure and acidic drinks.

### 11.3. Dry Mouth and Breath

Measure unstimulated salivary flow at baseline and annually (the annual interval is a pragmatic default extrapolated from general xerostomia monitoring rather than an evidence-derived figure); manage xerostomia with hydration counselling, xylitol-containing stimulants (which also reduce cariogenic bacteria), and saliva substitutes. For “Ozempic breath”, address the modifiable contributors—tongue cleaning, hydration, oral hygiene—and reassure that it often improves with adjustment [Grade B].

- Hydration counselling targeting adequate daily fluid intake (patients often drink markedly less) [20,21].
- Tongue cleaning and oral hygiene reinforcement to address volatile sulphur compound production [19].

### 11.4. Periodontal Care

Provide standard periodontal therapy and maintenance according to established risk-based standards, independent of incretin use. Any periodontal benefit of incretin therapy remains a hypothesis [12]; clinicians should not expect, or counsel patients to expect, periodontal improvement as a consequence of the drug, and should not assume that incretin therapy substitutes for periodontal treatment. Communicate to prescribers that periodontal therapy itself improves glycaemic control (HbA1c~0.4%), supporting integrated care [Grade B].

### 11.5. Implant Therapy

Optimise glycaemic control before implant placement (target HbA1c ≤ 7%); regard GLP-1 RA use as a possible—not proven—favourable modifier; intensify peri-implant maintenance in obese and diabetic patients [Grade C].

### 11.6. Sedation Safety

For procedures requiring deep sedation or general anaesthesia, coordinate with anaesthesia and follow current multi-society guidance, which now favours risk-stratified management over routine drug cessation [Grade B].

### 11.7. Interdisciplinary Communication

Refer back to the prescriber when dental findings suggest frequent severe vomiting (progressive erosion), new significant xerostomia, or rapidly progressive caries, any of which may prompt antiemetic optimisation or dose review [Grade C].

## 12. Limitations of the Current Evidence

Several limitations constrain the conclusions of this review and should be stated explicitly. First, dedicated human trials with oral health endpoints are essentially absent; most clinical inference is indirect, drawn from metabolic trials not designed to assess the mouth. Second, much of the mechanistic evidence for direct oral effects is preclinical (in vitro and rodent), and receptor expression demonstrated in animal salivary glands cannot be assumed to translate quantitatively to humans at therapeutic drug levels. Third, the patient-facing phenomena (“Ozempic mouth/teeth/breath/face”) are described mainly in narrative reviews, case series, and pharmacovigilance reports rather than in peer-reviewed prospective trials, and have been treated here as hypothesis-generating. Fourth, where benefits are reported (periodontal, peri-implant), confounding between direct receptor effects and indirect glycaemic improvement is rarely disentangled. Fifth, this is a narrative, not systematic, review and is subject to selection bias in the literature cited. Finally, the multi-receptor pipeline is evolving rapidly; trial figures cited here (e.g., for CagriSema and retatrutide) reflect data available to mid-2026 and will be superseded. Readers should therefore weigh the evidence accordingly: although the direction of the evidence is encouraging, its overall strength remains modest.

## 13. Research Agenda

The evidence gaps identified throughout this review translate into a concrete set of research priorities. Table 4 outlines the highest-yield questions and the study designs best suited to answer them, ranked by priority, and is aligned with the specific uncertainty identified in the preceding sections.

## 14. Conclusions

Incretin-based therapy—from the established GLP-1 mono-agonists and the dual agonist tirzepatide to an emerging frontier of triple agonists, glucagon co-agonists, amylin combinations, and GIP-antagonist hybrids—has reshaped metabolic medicine. The oral cavity is a plausible direct target of these drugs and a practical site for monitoring their tolerability. According to current evidence, incretin therapy may plausibly contribute to dry mouth, dental erosion, caries risk, and halitosis through recognised adverse effects, namely, nausea, vomiting, reduced fluid and food intake, delayed gastric emptying, and possible salivary changes; the patient-recognised phenomena of “Ozempic mouth”, “breath”, and “face” map onto these mechanisms. The incidence, severity, reversibility, and drug-specific causality of these effects remain insufficiently quantified; any periodontal benefit remains a hypothesis derived largely from preclinical and indirect data, while the net skeletal effect on the alveolus, balancing any direct receptor effect against the bone consequences of rapid weight loss, remains uncertain. These uncertainties do not diminish the practical preventive message: the irreversible component of any oral harm is avoidable with timely dental care.

The responsible conclusion is a measured one. The mechanistic and preclinical case is encouraging; the human evidence remains limited. Dental practitioners should engage actively, taking a medication history, preventing the irreversible hard-tissue damage that no later intervention can undo, managing dry mouth and breath, and coordinating sedation and prescriber communication, while neither overstating benefit nor dismissing patient concerns that have a genuine biological basis. The field’s most pressing need is straightforward: trials that place the mouth among their endpoints. Until then, careful clinical attention and honest communication remain the most appropriate standard of care for these patients.

## Figures and Tables

**Table 1:** Mechanism-based taxonomy of incretin and incretin-adjacent agents, organised by receptor target, with development status, oral health evidence status (reported separately so that the absence of oral data is explicit rather than conflated with regulatory maturity), and notes relevant to oral biology.

Receptor Target/Combination	Representative Agent(s)	Status	Oral Health Evidence Status	Notes Relevant to Oral Biology
GLP-1 (mono-agonist)	Semaglutide, liraglutide, dulaglutide, exenatide; oral semaglutide; oral small-molecule (orforglipron)	Approved	Direct oral evidence available (only class with any)	Best-characterised oral evidence; GLP-1R present in salivary gland (rodent), periodontal ligament, bone
GLP-1/GIP (dual agonist)	Tirzepatide	Approved	No direct oral data; inferred from receptor expression	Established: GIPR detected in rodent salivary tissue and bone. Oral relevance: hypothetical only—no clinical oral data; receptor presence does not imply an oral effect
GLP-1/GIP/glucagon (triple agonist)	Retatrutide	Phase 3 (TRIUMPH)	No oral data; entirely speculative	Established: highest weight loss to date (~24%, phase 2); glucagon agonism adds thermogenesis. Oral relevance: speculative—a higher GI/emetic burden is anticipated but unconfirmed, and no oral data exist
GLP-1/glucagon (dual co-agonist)	Survodutide, mazdutide	Phase 3	No oral data; entirely speculative	Established: glucagon-receptor agonism with hepatic and thermogenic effects. Oral relevance: none demonstrated—no oral data
GLP-1 RA + GIP antagonist (bispecific)	Maridebart cafraglutide (“MariTide”)	Phase 3	No oral data; entirely speculative	Established: mechanism distinct from tirzepatide (GIP blockade, not agonism). Oral relevance: unknown—no oral data
Amylin analogue (mono)	Cagrilintide; novel amylin monotherapies	Phase 2/3	No oral data; bone effect hypothesised only	Established: acts via amylin/calcitonin-receptor signalling. Oral relevance: hypothetical—a calcitonin-receptor-mediated bone effect is postulated but unproven; no oral data
GLP-1 + amylin (combination)	CagriSema (semaglutide + cagrilintide)	Phase 3 (REDEFINE)	No oral data; receptor presence noted, effect unstudied	Established: ~15.7–22.7% weight loss across REDEFINE trials; amylin receptors detected in oral mucosa/trigeminal system. Oral relevance: theoretical only—receptor presence is not evidence of an oral clinical effect
GLP-1/amylin (unimolecular dual)	Amycretin (oral & subcutaneous)	Phase 3 (entering)	No oral data; entirely speculative	Single molecule engaging both receptors; up to ~13–24% in early data. Oral relevance: speculative—no oral data
GIP (mono-agonist/antagonist, investigational)	Various preclinical	Preclinical stage	No oral data; role undefined	Role of isolated GIPR modulation in oral tissue undefined
GLP-1/GIP/NPY2R	Triple agonist peptide; not disclosed	Phase 2 (entering)	No oral data; relevance speculative	Relevance of NPY2R to salivary gland function speculative

Note: This taxonomy is organised by receptor target rather than brand name; a summary of approved-agent dosing is deliberately omitted, being available in standard references, in favour of a mechanism-first framework. Status reflects data available to mid-2026 and is subject to change.

**Table 2:** Evidentiary basis for the principal oral and facial phenomena attributed to incretin therapy. Categories: DH, direct human data; PV, pharmacovigilance/spontaneous-report signal; PC, preclinical (in vitro/animal) mechanism; EX, extrapolation from the general xerostomia/erosion/halitosis literature; EC, expert commentary or media/patient report.

Phenomenon	Best Available Supporting Evidence	Category
Dry mouth/xerostomia (“Ozempic mouth”)	Disproportionality signal in spontaneous-report databases; rodent salivary GLP-1R expression; reduced fluid intake; established downstream effects of hyposalivation. No direct human salivary flow-rate data.	PV, PC, EX
Enamel erosion/tooth damage (“Ozempic teeth”)	Trial-documented vomiting during escalation; well-established erosive effect of intrinsic gastric acid. The erosion mechanism is established; its incretin-specific incidence is not quantified.	EX, EC
Halitosis/belching (“Ozempic breath”)	Belching is a trial-reported effect (~7%); halitosis itself rests on mechanistic plausibility (reduced flow, ketosis) and commentary, with no dedicated peer-reviewed data.	EC, EX
Coated tongue	Described in the dental literature; mechanistically consistent with reduced salivary flow and impaired tongue cleansing, though not yet quantified in prospective studies.	EC, EX
Severe hyposalivation (case reports)	Small case series describing onset ~4 weeks after initiation; hypothesis-generating only.	DH (low-level), EC
Periodontal benefit	In vitro and animal periodontitis models plus a single clinical study; no HbA1c-controlled RCT with periodontal primary endpoints. Remains a hypothesis.	PC
Facial soft-tissue change (“Ozempic face”)	Predictable consequence of large-magnitude weight loss (not incretin-specific); dental relevance limited to prosthesis fit and soft-tissue support. Occlusal/jaw-function effects are hypothesised only.	EX, EC

**Table 3:** Summary of clinical recommendations for dental practitioners managing patients on incretin-based therapies, with GRADE-informed strength of evidence (A, randomised controlled trial evidence; B, observational/consensus with mechanistic support; C, expert opinion or extrapolation).

Domain	Key Action	Evidence Basis/Rationale	Grade
Medication history	Record incretin use, dose, nausea/vomiting/belching at every visit	Rationale: oral consequences cluster early and patients seldom disclose use; consensus/expert basis (EC).	C
Caries/erosion	5000 ppm fluoride; varnish; post-emesis water or bicarbonate rinse; defer brushing 30–60 min	Basis: erosion driven by treatment-emergent vomiting (a documented drug effect) acting through the established erosive mechanism of intrinsic acid; preventive measures extrapolated from general erosion guidance. Grade B reflects this mechanistic anchor plus indirect trial data, not analogy alone (EX, EC).	B
Dry mouth	Annual sialometry; xylitol stimulants; saliva substitutes; hydration	Basis: pharmacovigilance disproportionality signal and rodent salivary receptor data, plus reduced fluid intake as a drug-related driver; the sialometry interval is extrapolated from general xerostomia practice. Grade B reflects the mechanistic/indirect support (PV, PC, EX).	B
Breath	Tongue cleaning, hydration, hygiene; reassure on adjustment	Basis: belching is a trial-reported drug effect; halitosis management is extrapolated from the physiological halitosis literature but anchored in the same mechanism (reduced flow, delayed emptying). Grade B reflects this mechanistic link rather than analogy alone (EC, EX).	B
Periodontal	Standard therapy + maintenance; communicate HbA1c benefit to prescriber	Basis: preclinical periodontal benefit only; no HbA1c-controlled RCT. Maintenance follows established standards (PC, EX).	B
Implants	HbA1c ≤ 7% pre-op; GLP-1 RA a possible favourable modifier; intensified maintenance	Basis: preclinical osseointegration signal; implant benefit unproven, glycaemic target extrapolated (PC, EX).	C
Sedation	Follow current multi-society guidance; risk-stratified, not routine cessation	Basis: multi-society perioperative consensus guidance (EC).	B
Communication	Report erosion/xerostomia/caries to prescriber; prompt antiemetic or dose review	Rationale: links dental findings to prescriber-side mitigation; expert opinion (EC).	C

**Table 4:** Proposed research agenda for incretin therapies and oral health, ranked by priority, with the key unanswered question and a suggested study design for each.

Priority	Question	Suggested Design	Linked Uncertainty (Section)
1 (High)	Do GLP-1 RAs reduce periodontitis independent of glycaemic control?	RCT vs. active comparator, HbA1c-matched, periodontal endpoints primary	Section 5/Section 11.4 (periodontal benefit unproven; no HbA1c-matched RCT)
2 (High)	What is the true incidence/severity of xerostomia and its salivary basis?	Prospective cohort with sialometry and salivary proteomics over 24 months	Section 4 (no direct human salivary flow data)
3 (High)	What is the net caries/erosion incidence in incretin users?	Nested case–control within large obesity/T2DM cohort	Section 7 (erosion mechanism established; incidence unquantified)
4 (Moderate)	Do GLP-1 RAs improve dental implant osseointegration?	Prospective multicentre cohort: bone–implant contact, survival, peri-implantitis	Section 9/Section 11.5 (preclinical only; no implant outcome trials)
5 (Moderate)	How do multi-receptor agents (triple, amylin, GIP-antagonist) affect oral tissue?	Oral-health substudies embedded in ongoing Phase 3 programmes	Section 3.2 and Section 3.3 (no oral data for emerging classes)
6 (Moderate)	Does incretin therapy remodel the oral microbiome meaningfully?	Longitudinal shotgun metagenomics, pre/post initiation	Section 8 (microbiome effect plausible but unproven)
7 (Moderate)	Is “Ozempic breath” real, and what drives it?	Controlled halitosis study with volatile sulphur compound measurement and belching diary	Section 6 (halitosis rests on plausibility/commentary)

## Data Availability

This study did not generate new primary datasets.

## Abbreviations

The following abbreviations are used in this manuscript: GLP-1, glucagon-like peptide-1; GLP-1R, glucagon-like peptide-1 receptor; GLP-1 RA, glucagon-like peptide-1 receptor agonist; GIP, glucose-dependent insulinotropic polypeptide; GIPR, GIP receptor; T2DM, type 2 diabetes mellitus; HbA1c, glycated haemoglobin; DPP-4, dipeptidyl peptidase-4; ASA, American Society of Anesthesiologists; GRADE, Grading of Recommendations, Assessment, Development and Evaluations; GI, gastrointestinal; NPY2R, neuropeptide Y receptor 2.
